# The Prevalence and Clinical Presentation of Abdominal Tuberculosis in Patients With Acute Surgical Abdomen: A Prospective Observational Study

**DOI:** 10.7759/cureus.89732

**Published:** 2025-08-10

**Authors:** Nalini Singh, Swaroop Sanat Sahu, Kisslay Raj

**Affiliations:** 1 General Surgery, Rajendra Institute of Medical Sciences, Ranchi, IND; 2 General Surgery, Rajendra institute of Medical Sciences, Ranchi, IND

**Keywords:** abdominal tuberculosis, acute abdomen, antitubercular therapy, cbnaat, diagnostic accuracy, histopathology

## Abstract

Background and objective

The diagnosis of abdominal tuberculosis (ATB) poses significant difficulty in the context of acute abdomen cases, especially in regions where it is endemic. This study aimed to determine the frequency and clinical manifestations of ATB among adults with an acute surgical abdomen at a tertiary care center in Ranchi, India, focusing on the means of arriving at the diagnosis and subsequent treatment results.

Methods

Our study involved 60 patients with acute abdominal symptoms and was conducted during the year 2023-2024 at the Rajendra Institute of Medical Sciences, Ranchi. We collected demographic data along with imaging studies such as X-ray, ultrasound, CT scan, acid-fast bacilli (AFB) smear, cartridge-based nucleic acid amplification test (CBNAAT), intraoperative findings, histopathological examination (HPE), and symptomatology to perform relevant and timely diagnosis. The association between clinical symptoms of the patients and ATB was assessed using the chi-square test.

Results

The confirmation rate of ATB was five (8.3%) among the patients. A significant association was observed with fever and weight loss (p<0.01), although all patients did report some degree of abdominal pain. HPE showed high accuracy with 90% sensitivity and 95% specificity. For CT imaging, sensitivity and specificity were reported at 70% and 85%, respectively, while ultrasound sensitivity lagged at approximately 60%. Anti-tuberculosis therapy (ATT) had demonstrable benefits for all ATB patients. Common intraoperative findings involved lymphadenopathy along with ascites.

Conclusions

The data indicate that ATB was present in five (8.3%) cases, manifesting as acute abdomen presenting surgically; we identified weight loss and fever as main symptoms that contributed to our diagnostic approach, leading us to diagnose it more effectively than would otherwise be possible in unexplained abdominal pain cases. HPE-based imaging tests performed alongside appropriate medications showed excellent results in endemic areas.

## Introduction

Abdominal tuberculosis (ATB) refers to an extrapulmonary form of tuberculosis infection that has an economic burden in areas such as India, and it accounts for 5-15% cases of acute surgical abdomen [1]. It has been dubbed the "notable imitator" due to the vague symptoms of abdominal pain, appetite loss, fevers, and distension alongside weight loss, all featuring similar symptoms like appendicitis or Crohn's disease, and even cancer. The inflammation and/or infection of these structures - involving the gastrointestinal tract, lymph nodes, and peritoneum - further adds to the difficulty both from a clinical and diagnostic perspective in emergencies such as severe abdominal pain, where patients may require prompt intervention to avoid complications such as bowel obstruction or perforation [2].

There exists a global discrepancy regarding ATB prevalence, with underdiagnosis being more pronounced in countries with lower incidences of tuberculosis. This is likely due to people's lack of clinical suspicion. Low-income countries tend to suffer from widespread TB and HIV/AIDS, alongside malnutrition, thereby having a higher likelihood of contracting ATB subsequently. Meanwhile, multi-drug resistant TB strains and HIV/AIDS are exacerbating pre-existing multi-drug resistance, thereby contributing to the medical burden globally, resulting in a rising incidence among immunocompromised patients. In India, for instance, ATB affecting the ileocecal region tends to mimic other intra-abdominal pathologies, thereby presenting with an obstructive picture or mass effect [3,4].

ATB's subtle onset and absence of distinct symptoms make its diagnosis particularly challenging [4]. Imaging modalities such as ultrasound and CT may reveal features such as intestinal thickening, ascites, or lymphadenopathy, but these findings are nonspecific and should be interpreted with caution. Although histopathological examination (HPE) is considered the gold standard, it is not feasible in emergencies. While molecular assays like polymerase chain reaction (PCR) and cartridge-based nucleic acid amplification test (CBNAAT) improve detection, their limited availability in resource-limited areas makes prompt diagnosis difficult. Risk factors for ATB include travel to endemic regions, known exposure to tuberculosis, and immunosuppression [5,6].

Anti-tuberculosis therapy (ATT) with ethambutol, rifampin, pyrazinamide, and isoniazid is administered for about six to nine months, followed by surgical interventions for complications such as intestinal obstruction. Serious consequences like sepsis can often be avoided with early diagnosis and treatment. Even though tuberculosis is endemic in eastern India, data on ATB in this area remain limited [7,8,9]. Based on the hypothesis that a systematic strategy enhances both diagnosis and clinical outcomes, this study aimed to assess the prevalence, clinical characteristics, diagnostic approaches, and treatment outcomes of ATB in patients presenting with an acute surgical abdomen at the Rajendra Institute of Medical Sciences, Ranchi.

## Materials and methods

Study design

This was an observational and longitudinal study, examining patients diagnosed with acute abdomen and focusing particularly on ATB to assess their clinical features, diagnostic accuracy, and their intraoperative findings, as well as their outcomes. We prospectively recorded data during the routine course of the illness.

Study setting and duration

The study was conducted in the General Surgery Department of the Rajendra Institute of Medical Science, Ranchi, which is a tertiary care teaching hospital in India. The hospital has modern diagnostic and surgical facilities.

Study population

The participants included all adult patients aged between 18 and 70 years who presented to the emergency department with symptoms of acute abdomen and subsequently underwent diagnostic laparoscopy or laparotomy. Clinical data were collected for each patient, including those who had a history of pulmonary or extrapulmonary tuberculosis.

Inclusion criteria

We included patients in the age range of 18-70 years who had some form of abdominal pain. People with exposed surgical exploration to assess intra-abdominal pathology after evaluation and treatment in the emergency room or ward level surgery were included. Some patients also presented with acute gastrointestinal symptoms with a known history of tuberculosis. Patients were only included in the trial if they provided informed written consent to participate and receive ATT. No deaths related to ATT were noted. The frequently noted intraoperative findings were lymphadenopathy and/or ascites.

Exclusion criteria

Elderly patients aged 70 years and above, as well as those under 18 years old, were excluded. Additional reasons for exclusion included acute diabetes, along with other systemic ill conditions, and escalating non-operable severe endocrine metabolic diseases. Expectant mothers or any individual presenting with acute trauma to their stomach region were also excluded. Patients with advanced-stage metastasis and those with a history of pronounced tumoral pathologies were also excluded.

Sampling technique and sample size

Convenience sampling was used to recruit patients for the study [7]. All consecutive patients who showed up at the emergency departments or surgical units during the study period and satisfied the inclusion requirements were enrolled. Consecutive recruitment was used to minimize selection bias and to ensure that the sample realistically reflected the spectrum of acute surgical abdominal conditions seen in clinical practice. Assuming a 4% prevalence of abdominal tuberculosis among acute abdomen cases, the sample size was calculated using the formula below:

$$n = \frac{Z^2 pq}{d^2} \tag{1}$$

Where Z = 1.96 (95% confidence), p = 0.04, q = 0.96, and d = 0.05, resulting in a minimum of 60 samples. This made sure there was enough power to find statistically significant correlations between histopathological, radiological, and clinical characteristics. To improve statistical robustness and assist subgroup analyses, all eligible patients who presented throughout the research period were included, even though 60 was the determined criterion.

**Figure 1:**
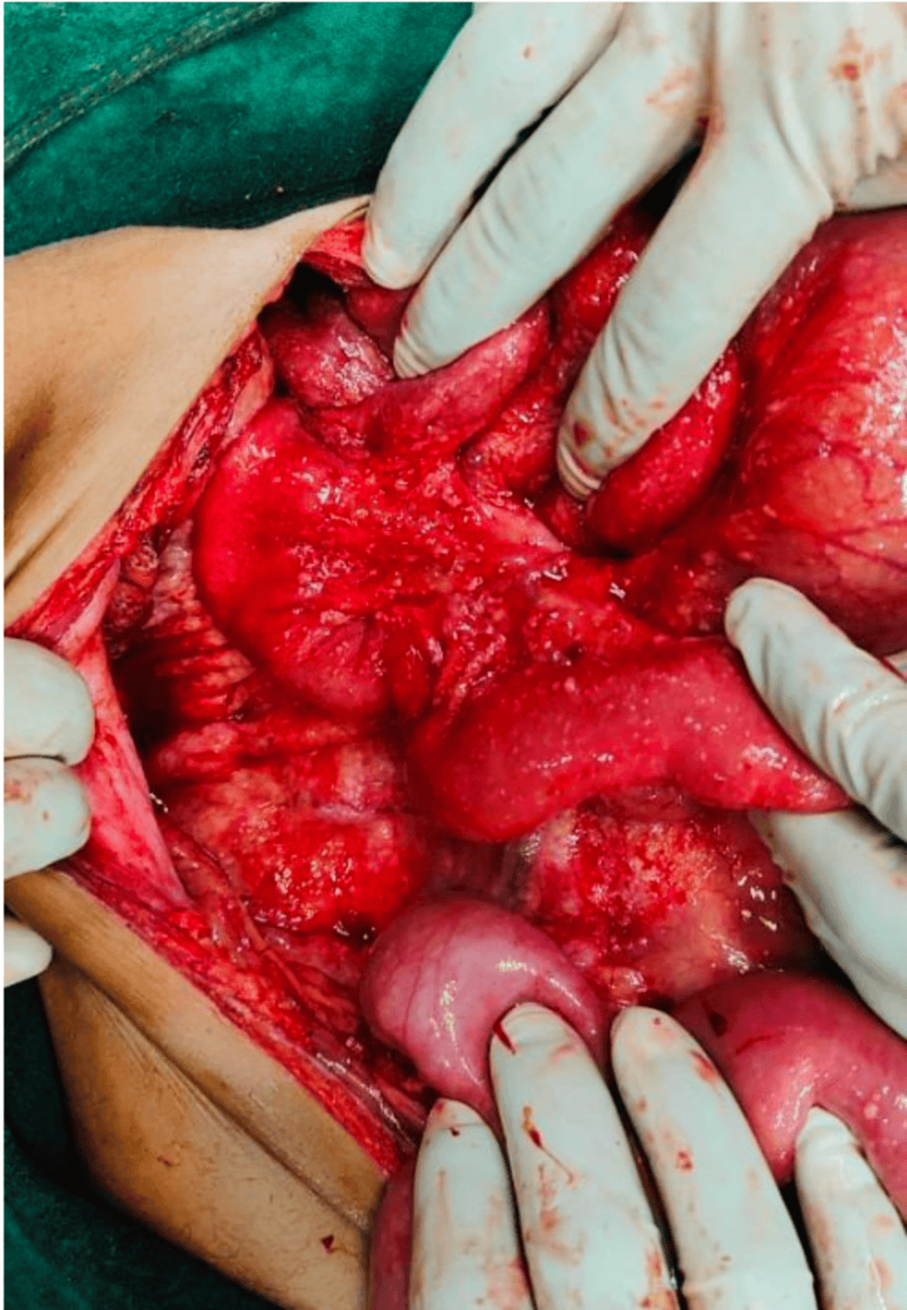
Intraoperative image - 1 An intraoperative image displaying extensive fibrous adhesions involving the abdominal wall and small intestine loops is frequently observed in patients who have had previous surgeries or who have long-term inflammatory diseases such as tuberculosis or perforation-related peritonitis Image credit: Kisslay Raj

**Figure 2:**
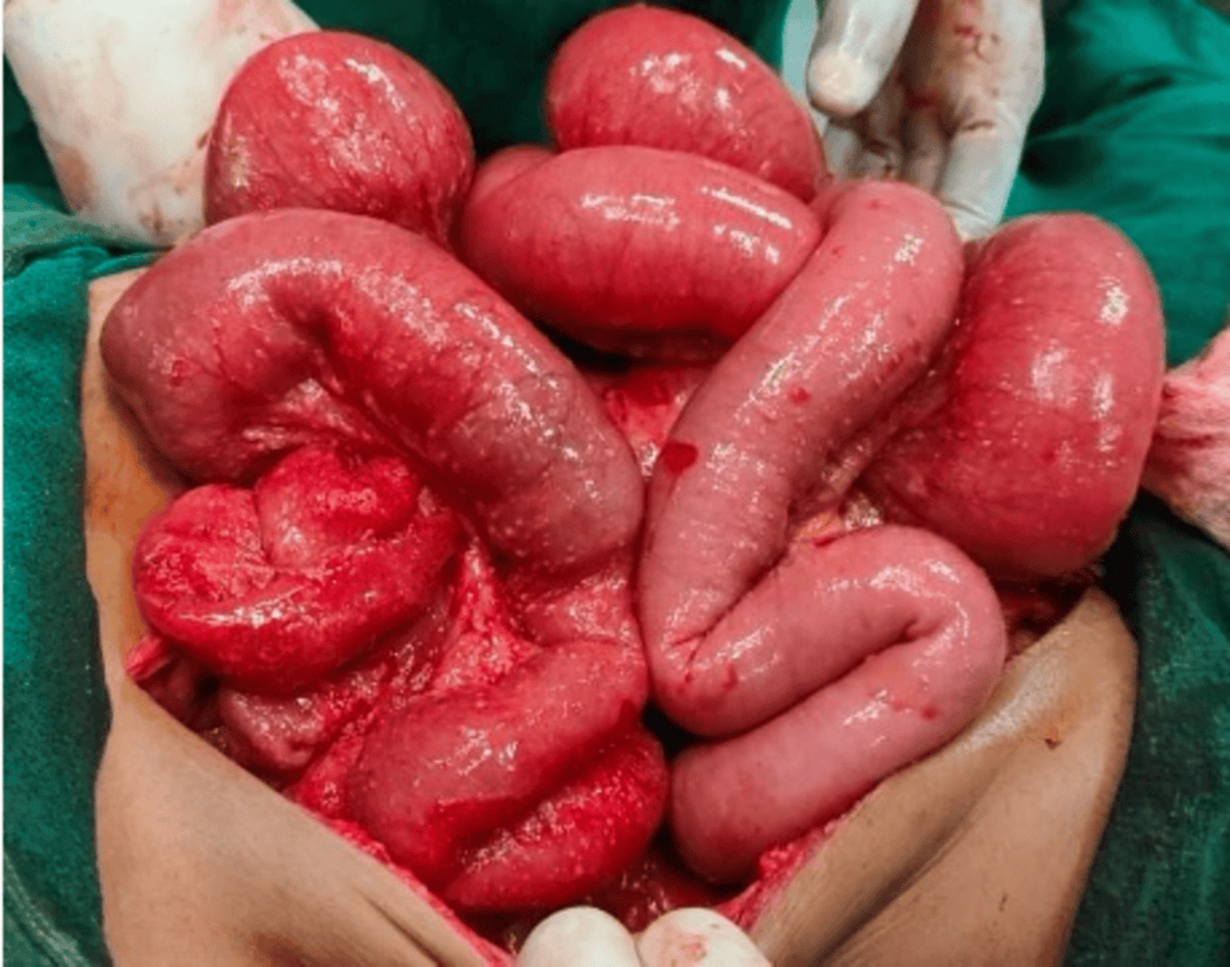
Intraoperative image - 2 Multiple tiny, homogeneous, white nodules scattered over the small intestine's surface are shown on the intraoperative imaging; these are indicative of granulomatous infections or abdominal tuberculosis Image credit: Kisslay Raj

Based on intraoperative and histological findings, patients were categorized into four groups according to their postoperative diagnosis. Volvulus, hernias, malignancies, and adhesions with dense fibrous bands observed after surgery were among the obstructive causes (Figure 1). Amoebiasis, intestinal perforation, and abdominal tuberculosis, which often present as characteristic miliary nodules on the serosal surface of the intestines, were among the infectious causes (Figure 2). Patients with tubercular perforation, typhoid, and peptic ulcers comprised the perforation peritonitis group. Mesenteric ischemia, abdominal trauma, and neoplasms were classified as other causes.

Data collection

A standardized proforma was used to record patient data, capturing important clinical and investigative information. Demographic data included socioeconomic status, age, and sex. Clinical information encompassed the onset and duration of abdominal pain, associated symptoms, and relevant physical examination findings. A thorough medical history was taken, focusing on prior tuberculosis, any contact with TB patients, and coexisting medical conditions. The investigations included various imaging studies such as contrast-enhanced CT, plain abdominal X-ray, and ultrasonography [5]; biochemical parameters, which included liver and renal function tests (LFT and RFT); hematological checks such as complete blood count (CBC), erythrocyte sedimentation rate (ESR), and C-reactive protein (CRP) [7]. Intraoperative findings were documented in detail, focusing on the type and extent of pathology and the organs involved. Histopathological examination of biopsy specimens was performed to confirm the diagnoses, particularly ATB [3,9]. Patient outcomes, including duration of hospital stay, postoperative complications, and mortality, were also documented. All information was anonymized and systematically added into a secure master database, with regular validation to ensure accuracy and completeness.

Statistical analysis

In this study, clinical characteristics and outcomes were summarized using descriptive statistics. For comparing continuous data, independent t-tests were employed, while categorical data were assessed through chi-square tests. ATB prevalence and subgroup comparisons were computed. A p-value of less than 0.05 was used as the cutoff value for statistical significance.

Ethical considerations

The study conformed to the ethical guidelines set by the Institutional Ethics Committee (IEC) of Rajendra Institute of Medical Sciences (RIMS), Ranchi, and followed the principles described in the Declaration of Helsinki. The IEC provided approval for the study (approval no. 266, dated 10.08.2024). Written informed consent was obtained from all participants before enrollment, which guaranteed voluntary participation and confidentiality. All procedures conducted within the study were limited to routine diagnostic and therapeutic care, thus posing no incremental risk to patients beyond what is standard. Confidentiality of data was ensured; all information retrieved remained confidential and was exclusively utilized for research analysis.

## Results

Demographic and clinical data

A total of 60 adult patients, aged 18-70 years, presenting with acute abdomen in a tertiary care hospital were included in this study. The majority of the study population was young: 20 (33.3%) were in the age group of 18-30 years, 18 (30.0%) were in the age group of 31-40 years, 12 (20.0%) were in the age group of 41-50 years, and 10 (16.7%) were over the age of 50 years. The male-to-female ratio was 40:20 (66.7%:33.3%), showing a preponderance of males, which could have been due to differences in risk factors or gender-related healthcare-seeking behavior. ATB was diagnosed in five (8.3%) patients by histopathology, CBNAAT, or acid-fast bacilli (AFB) smear. All 60 (100%) patients had abdominal discomfort at presentation; 12 (20.0%) had weight loss, 18 (30.0%) had abdominal distension, 15 (25.0%) had bowel disturbances, 25 (41.7%) had fever, and 40 (66.7%) had nausea and vomiting. Abdominal tenderness was found on clinical examination in 55 (91.7%), guarding in 30 (50.0%), rigidity in 28 (46.7%), rebound tenderness in 20 (33.3%), and tachycardia in about 60% of the patients, which was a sign of systemic stress or peritoneal irritation. These results indicated the varied presentations of acute abdomen, with ATB representing a significant subgroup warranting particular diagnostic attention (Table 1).

**Table 1:** Demographic characteristics, clinical presentations, and examination findings in patients with acute abdomen, highlighting ATB cases ^*^P<0.05 (statistically significant) For categorical variables, chi-square testing yielded p-values ATB: abdominal tuberculosis

Parameters	Category	Number	Percentage (%)	P-value
Age groups (years)	18-30	20	33.3	0.87
	31-40	18	30.0	0.87
	41-50	12	20.0	0.87
	>50	10	16.7	0.87
Gender	Male	40	66.7	0.03
	Female	20	33.3	0.03
ATB confirmed	Positive	5	8.3	0.01^*^
Symptoms	Abdominal pain	60	100	0.001
	Nausea/vomiting	40	66.7	0.001
	Fever	25	41.7	0.005
	Abdominal distension	18	30.0	0.22
	Bowel disturbances	15	25.0	0.22
	Weight loss	12	20.0	0.22
Clinical signs	Abdominal tenderness	55	91.7	0.001
	Guarding	30	50.0	0.01^*^
	Rigidity	28	46.7	0.01^*^
	Rebound tenderness	20	33.3	0.05

Vital signs and laboratory parameters

The physiological status of patients at presentation was assessed using vital signs. The systolic blood pressure was moderately stable, with an average of 120 ± 15 mmHg. The mean pulse rate was 98 ± 20 beats per minute, indicating mild tachycardia that was probably brought on by pain, anxiety, or systemic illness. Despite systemic stress, the average oxygen saturation was 96 ± 2%, indicating adequate respiratory function, and the mean body temperature was 37.8 ± 0.8°C, suggesting potential inflammatory or infectious processes. The results of laboratory tests showed mild anemia (mean hemoglobin: 10.8 ± 1.5 g/dL), an elevated total leukocyte count (12,000 ± 3,000 cells/mm³), and an elevated ESR (40 ± 10 mm/hr), all of which were suggestive of infection or systemic inflammation. The serum protein levels at the lower end of normal (6.0 ± 1.0 g/dL) and albumin (3.5 ± 0.5 g/dL) could have indicated acute-phase responses or underlying malnutrition (Table 2).

**Table 2:** Laboratory results and vital signs at presentation, indicating inflammatory markers and systemic condition ESR: erythrocyte sedimentation rate; SD: standard deviation; WBC: white blood cells

Parameter	Mean ± SD	Units	Reference range
Systolic blood pressure	120 ± 15	mmHg	-
Heart rate	98 ± 20	beats/min	-
Body temperature	37.8 ± 0.8	°C	-
Blood oxygen level	96 ± 2	%	-
Hemoglobin concentration	10.8 ± 1.5	g/dL	12-16
Total WBC count	12,000 ± 3,000	cells/mm³	4,000-11,000
ESR	40 ± 10	mm/hr	0-20
Serum protein	6.0 ± 1.0	g/dL	6.0-8.0
Albumin	3.5 ± 0.5	g/dL	3.5-5.0

Diagnostic outcomes

Of the 60 patients enrolled, ATB was diagnosed in five (8.3%). With 90% sensitivity and 95% specificity, HPE was the most accurate diagnostic method, accurately confirming ATB (Table 3). Among 40 tissue samples tested, CBNAAT detected TB in five (12.5%) cases, while AFB smear was positive in four (10%) cases. In ascitic fluid samples (n = 20), AFB smear was positive in three (15%) cases, and CBNAAT in four (20%), reflecting the paucibacillary nature of ATB. Adenosine deaminase (ADA) levels were ≥40 U/L in 12 (60%) samples, and elevated lactate dehydrogenase (LDH) was observed in five (25%), further supporting the diagnosis (Table 4).

**Table 3:** ATB diagnostic modalities, displaying specificity, sensitivity, and positive and negative predictive values ATB: abdominal tuberculosis; CBNAAT: cartridge-based nucleic acid amplification test; CT: computed tomography; HPE: histopathological examination; NPV: negative predictive value; PPV: positive predictive value

Modality	Sensitivity (%)	Specificity (%)	PPV (%)	NPV (%)
Ultrasound	60	80	30	93
CT scan	70	85	40	95
HPE	90	95	83	97
CBNAAT	75	90	60	94

**Table 4:** Microbiological and ascitic fluid analysis results for the diagnosis of ATB ADA: adenosine deaminase; AFB: acid-fast bacilli; ATB: abdominal tuberculosis; CBNAAT: cartridge-based nucleic acid amplification test; LDH: lactate dehydrogenase

Test	Positives (N)	Percentage (%)
Tissue/sample (n = 40)		
AFB smear	4	10.0
CBNAAT	5	12.5
Both AFB and CBNAAT	3	7.5
Negative	28	70.0
Ascitic fluid (n = 20)		
AFB smear	3	15.0
CBNAAT	4	20.0
ADA (≥40 U/L)	12	60.0
Elevated LDH	5	25.0

Radiological imaging played a critical role in the diagnostic process. Chest X-rays were normal in 49 (81.7%) patients; however, they showed pulmonary consolidation in eight (13.3%), and a miliary pattern in three (5.0%), suggesting possible pulmonary involvement (Table 5). Abdominal X-rays were normal in 40 (66.7%) patients, while 15 (25.0%) showed evidence of obstruction, and five (8.3%) showed signs of perforation. Ultrasound findings included ascites in 20 (33.3%) patients, abdominal mass in 12 (20.0%), omental thickening in 10 (16.7%), lymphadenopathy in eight (13.3%), and normal results in 25 (41.7%). Ultrasound demonstrated a sensitivity of 60% and specificity of 80% in diagnosing ATB. CT scans were performed in 30 (50%) patients and showed 70% sensitivity and 85% specificity. Findings included ascites in 12 (40.0%) patients, abdominal mass in 10 (33.3%), bowel wall thickening in eight (26.7%), lymphadenopathy in six (20.0%), and normal findings in 10 (33.3%). Non-ATB diagnoses included infectious causes (e.g., peritonitis), obstructive pathologies (e.g., intestinal obstruction, adhesions) in 25% of cases, perforations such as perforated peptic ulcer in 20%, and other causes (e.g., inflammatory conditions and vascular ischemia) confirmed intraoperatively or via imaging (Table 6).

**Table 5:** Radiological results from CT, ultrasound, and chest and abdominal X-rays CT: computed tomography

Investigative measure	Finding	Number	Percentage (%)
Chest radiograph (X-ray)	Normal	49	81.7
	Consolidation	8	13.3
	Miliary pattern	3	5
Abdominal radiograph (X-ray)	Normal	40	66.7
	Obstruction	15	25
	Perforation	5	8.3
Ultrasound (n = 60)	Normal	25	41.7
	Ascites	20	33.3
	Abdominal mass	12	20
	Omental thickening	10	16.7
	Lymphadenopathy	8	13.3
CT scan (n = 30)	Normal	10	33.3
	Ascites	12	40
	Abdominal mass	10	33.3
	Bowel wall thickening	8	26.7
	Lymphadenopathy	6	20

**Table 6:** Symptoms comparison between TB-positive and TB-negative patients ^*^P<0.05 (statistically significant)

Symptoms	TB-positive (n = 5)	TB-negative (n = 55)	P-value
Weight loss	4 (80.0%)	8 (14.5%)	0.01^*^
Fever	5 (100%)	20 (36.4%)	0.005^*^
Abdominal pain	5 (100%)	55 (100%)	0.22
Vomiting/nausea	3 (60.0%)	37 (67.3%)	0.22

Treatment and complications

All five ATB patients (100%) received ATT with standard regimens (isoniazid, rifampin, pyrazinamide, ethambutol) for six months, achieving symptom resolution in most cases by 12 weeks. Surgical intervention was required in 10 (16.7%) of the total cohort, primarily for obstructive or perforative complications. Specific surgical rates for ATB patients were one (20%). Intraoperative findings included ascites in four (80%) ATB patients, adhesions in three (60%), strictures in one (20%), perforations in one (20%), and mass lesions in two (40%), with ATB cases often showing omental thickening and lymphadenopathy. In cases with suspected or confirmed ATB requiring surgical intervention (primarily for obstruction or perforation), procedures included adhesiolysis alone (for peritoneal adhesions without significant bowel involvement), strictureplasty (for isolated or multiple short strictures to preserve bowel length), and resection with primary anastomosis (e.g., segmental small bowel resection with end-to-end anastomosis or limited ileocolic resection for ileocecal involvement).

Simple anastomosis without resection was not performed, as it was deemed unsuitable for diseased segments. In the ATB subgroup, two cases underwent resection anastomosis for obstructive strictures, one underwent strictureplasty, and the rest were managed conservatively with ATT after diagnostic exploration confirmed no acute complications necessitating resection. Surgical decisions prioritized conservative approaches where possible to minimize risks in malnourished patients. Overall, complications included intestinal obstruction in 10 (16.7%) patients and perforation in five (8.3%), managed either conservatively or surgically depending on severity and clinical context. Non-ATB patients, particularly those with appendicitis (28%) or perforation peritonitis (20%), had higher surgical intervention rates (approximately 45%). No mortality was reported in ATB cases, and complication rates did not significantly differ across age groups (p>0.05) (Table 7).

**Table 7:** Frequency of complications in the total cohort The p-values indicate a lack of significant differences ATB: abdominal tuberculosis

Complication	N (total cohort)	Percentage (%)	ATB cases (n = 5)	Non-ATB cases (n = 55)	P-value
Obstruction	10	16.7	1 (20%)	9 (16.4%)	0.22
Perforation	5	8.3	1 (20%)	4 (7.3%)	0.22
Surgical intervention	10	16.7	1 (20%)	9 (16.4%)	0.22
Mortality	0	0	0 (0%)	0 (0%)	-

ATB was identified in five (8.3%) acute abdomen cases, with fever and weight loss significantly correlated with ATB diagnosis (p<0.01). HPE (90% sensitivity, 95% specificity) and CBNAAT (75% sensitivity, 90% specificity) were key diagnostic tools, supported by radiological findings like ascites and lymphadenopathy. ATT was effective in all ATB cases, with low complication rates and no mortality. Non-ATB cases required more frequent surgical intervention. The study emphasizes the need for high clinical suspicion, particularly for fever and weight loss, and integrated diagnostic approaches for early identification and treatment of ATB in cases of various severe abdominal symptoms.

## Discussion

This prospective observational study from a TB-endemic region illustrates the significant burden and diagnostic challenges posed by ATB among acute abdomen patients. ATB was confirmed in five (8.3%) of 60 patients assessed, which is consistent with current literature: an incidence of 5-10% [10,11]. In line with previous findings, the cohort was primarily male (66.7%), with the largest age group being 18-30 years (33.3%), indicating a demographic at risk for tuberculosis because of increased exposure and travel [12,13]. Clinically, every patient had 100% abdominal discomfort when they first arrived, while fever (41.7%) and nausea/vomiting (66.7%) were also observed. Notably, fever and weight loss were significantly more frequent among ATB patients compared to non-ATB cases (100% vs. 36.4%, p = 0.005 and 80.0% vs. 14.5%, p = 0.01), highlighting their importance as red-flag symptoms [14].

Laboratory evaluations revealed elevated ESR and total leukocyte count TLC among patients with ATB, suggesting a consistent systemic inflammatory response. CBNAAT showed limited sensitivity in tissue (12.5%) and slightly higher in ascitic fluid (20%), consistent with the low bacterial load in ATB. Combining microbiological tests with ADA and histopathology improved diagnostic accuracy [15,16]. Radiologically, CT scan was performed in 30 out of 60 patients and showed more sensitivity (70%) and specificity (85%) compared to ultrasound (60%), and commonly revealed ascites (40.0%), lymphadenopathy (20.0%), and omental thickening (22.5%), which are classical features of peritoneal TB [15,16]. However, HPE remained the gold standard, which showed granulomatous inflammation in 100% of confirmed ATB cases, demonstrating the highest diagnostic yield [17].

Intraoperative findings included ascites (80.0%), adhesions (60.0%), and mesenteric lymphadenopathy (40.0%), which further reinforced the diagnosis of ATB and matched observations from prior studies [13]. Of the total patients, 16.7% required surgical intervention, with perforation (8.3%) and obstruction (16.7%) being the main indications. All confirmed ATB cases were managed with ATT, and most showed favorable responses, with a 100% survival rate and 80% reporting symptom resolution post-treatment, underscoring the effectiveness of standardized ATT regimens [17].

Regarding surgical management in ATB with bowel involvement, literature supports a conservative approach to minimize resection and preserve bowel function, given the systemic nature of the disease and frequent malnutrition in patients [18,19]. For obstructive strictures, strictureplasty (e.g., Heineke-Mikulicz technique) is often preferred over resection for multiple short lesions, as it avoids short bowel syndrome and has low recurrence rates [17]. However, in cases of long strictures, perforations proximal to strictures, or ileocecal masses, resection with primary anastomosis (e.g., segmental small bowel resection with end-to-end anastomosis or right hemicolectomy with ileocolic anastomosis) is more suitable, demonstrating low complication rates (e.g., anastomotic leak: <5%) in stable patients without sepsis [20]. Simple bypass anastomosis is largely obsolete due to higher risks of blind loop syndrome and persistent disease [21]. In emergencies with sepsis or peritonitis, a staged approach, initial resection with diversion (e.g., ileostomy) followed by delayed closure after ATT is recommended to reduce morbidity [22]. Systematic reviews indicate primary anastomosis is safe in non-septic obstructive cases, with outcomes superior to staged procedures in elective settings [10]. Our findings align with this, as surgical interventions in ATB cases focused on complication resolution while prioritizing ATT for primary treatment.

The study demonstrates that ATB should be considered in endemic locations when developing a differential diagnosis for acute abdomen, especially in young individuals with nebulous systemic symptoms. Early diagnosis and care are essential to avoid problems like stricture formation or perforation, which may call for surgery.

There are some limitations to the study. The small sample size, single-center design, and lack of long-term follow-up reduce the generalizability of its findings [15]. Operator dependency in ultrasound interpretation and limited microbiological sensitivity may also affect diagnostic accuracy [16]. Moreover, while CBNAAT has improved diagnostic timelines, its moderate sensitivity (12.5%) restricts it from being a standalone diagnostic tool in peritoneal TB [10,15].

Future investigations should focus on multicentric prospective studies with larger populations, standardized diagnostic algorithms, and integration of molecular techniques with imaging and histopathology. Early detection could also be improved by investigating economical screening methods and AI-assisted diagnostics in environments with limited resources [17]. ATB is still a significant and frequently overlooked cause of acute abdomen. This study provides some deep insights, especially into the role of histopathology, and supports a multimodal approach to diagnosing ATB. It emphasizes the need to maintain high levels of clinical suspicion in endemic areas. The majority of ATB cases can be considerably improved by early diagnosis and suitable therapy.

## Conclusions

ATB is still a key differential diagnosis in patients presenting with an acute abdomen, particularly in TB-endemic regions. A notable proportion of cases in our study were attributed to ATB, with fever and weight loss being the two most important clinical signs. Histopathology was the most accurate diagnostic method found in this study. No fatality was documented; early diagnosis and antitubercular treatment led to positive results. A systematic, multimodal approach is necessary in these patients to ensure better diagnostic accuracy and to direct prompt surgical intervention when necessary.
